# Advancing Precision in Post-mastectomy Chest Wall Radiotherapy: A Comparative Dosimetric Analysis of Volumetric-Modulated Arc Therapy (VMAT) and Intensity-Modulated Radiotherapy (IMRT) Based on Institutional Experience

**DOI:** 10.7759/cureus.38464

**Published:** 2023-05-02

**Authors:** Shiv S Mishra, Siddhartha Nanda, Manish K Ahirwar, Simran ., Swaroopa M Rath

**Affiliations:** 1 Radiation Oncology, All India Institute of Medical Sciences, Raipur, Raipur, IND; 2 Medicine, Srirama Chandra Bhanja (SCB) Medical College and Hospital, Cuttack, IND

**Keywords:** intensity modulated radiotherapy (imrt), volumetric modulated arc therapy (vmat), radiotherapy, breast cancer, post-mastectomy

## Abstract

Background: Post-mastectomy radiation therapy (PMRT) is an important component in the management of breast cancer patients who have undergone mastectomy. Intensity-modulated radiation therapy (IMRT) and volumetric-modulated arc therapy (VMAT) are two popular methods of delivering PMRT.

With IMRT, high radiation doses are directed at the tumor, while exposure to healthy tissue is kept to a minimum. VMAT, on the other hand, is a more advanced version of IMRT that allows for faster radiation dose delivery while maintaining precision. The complexity of the VMAT treatment planning and delivery process, on the other hand, may increase the risk of technical errors, which can reduce treatment effectiveness.

Studies have compared VMAT and IMRT in PMRT for breast cancer patients, but most have found no significant differences in treatment outcomes between the two methods. Individual patient factors such as treatment goals, available resources, and other characteristics may influence the choice between the two techniques.

Purpose: This prospective observational study aimed to compare the dosimetry of two cutting-edge modern radiotherapy techniques for post-mastectomy breast cancer patients receiving hypofractionated doses.

Methods: For 58 patients with breast cancer, 116 plans for radiotherapy treatment were generated by both VMAT and IMRT. To maintain the uniformity of contouring, every CT image was contoured by the same physician, and Radiotherapy Oncology Group (RTOG) contouring guidelines were strictly followed during contouring.

Results: Both techniques had comparable target volume coverage, but VMAT produced a significantly better conformity index than IMRT for both the left (0.71 vs. 0.65) and right (0.72 vs. 0.66) breasts (p-value < 0.05). VMAT plans had significantly higher low-dose spillage to the ipsilateral lung (V5Gy and V10Gy) but significantly lower high-dose spillage (V20Gy, V30Gy, and V40Gy) than IMRT plans (p-value < 0.05). Dmax and Dmean for the ipsilateral lung were comparable for both techniques. When compared to alternative treatment approaches, IMRT treatment plans were found to be more effective in minimizing radiation exposure to the heart for all patients with right-sided breast cancer, resulting in considerably lower levels of Dmean, V5Gy, V10Gy, V20Gy, and V35Gy. Plans for VMAT treatment were found to be significantly superior to left-side chest wall radiotherapy in terms of lower exposure to the heart for higher doses. IMRT plans, on the other hand, were successful in dramatically lowering the levels of Dmax that reached the spinal cord for both right- and left-sided breast cancers.

Conclusion: Apart from similar planning target volume (PTV) coverage to IMRT plans, VMAT produced significantly better conformity. VMAT plans have more low-dose spillage to normal tissues, while IMRT plans spare various organs at risk significantly better at lower doses in both right and left-sided breast cancer. VMAT was found to be better at sparing the heart (in left-sided breast cancer only) and ipsilateral lung at a high dose range. The best radiotherapy approach for breast cancer should be established on an individual basis, taking into account tumor laterality and the risk-benefit ratio.

## Introduction

Breast cancer constitutes 24.5% of all new cancer cases in women and 13.5% in both sexes globally [1]. Post-mastectomy radiotherapy (PMRT) is a proven treatment to significantly reduce the risk of local recurrence and improve survival [2-4]. There are various methods available for delivering PMRT to patients, and it can be challenging to determine the optimal technique among options such as three-dimensional conformal radiotherapy (3D-CRT), intensity-modulated radiotherapy (IMRT), and volumetric-modulated arc therapy (VMAT).

The conventional approach for delivering radiotherapy, called 3D-CRT, uses tangential beams to limit the dose to organs at risk (OARs). However, it often leads to poor radiotherapy plans [5]. VMAT and IMRT offer much better dose distribution and target coverage [6]. VMAT, in particular, is known to provide a better radiotherapy plan with a shorter treatment time than IMRT [7,8]. However, both techniques may increase low-dose spread to normal tissue [9].

VMAT significantly reduces treatment time by manipulating the multileaf collimators (MLCs) and dose rate during treatment. In contrast, the increase in treatment time and monitor units (MUs) in IMRT may have implications for tumor cell repair and repopulation [10,11].

Historically, conventional radiotherapy fractionation had been utilized for breast radiotherapy, but many randomized controlled trials have demonstrated that hypofractionated irradiation is non-inferior to conventionally fractionated irradiation in terms of survival and toxicity profile [12-15]. Hence, in our study, we have included the patients who are receiving hypofractionated radiotherapy. Our study aims to contrast the dosimetric aspects of the treatment regimens for this particular patient group using VMAT and IMRT.

## Materials and methods

Patient selection

A prospective observational study was conducted in our department from March 2019 to March 2021, which included 58 breast cancer patients who underwent modified radical mastectomy. The demographic and clinical characteristics of the enrolled patients are presented in Table 1.

**Table 1:** Demographic, clinical, and treatment characteristics of enrolled patients

Age (Years)	Right-sided breast cancer	Left-sided breast cancer	Total
≤40	12	6	18
40-50	10	4	14
>50	18	8	26
Gender			
Male	2	0	2
Female	38	18	56
Stage			
I	2	0	2
II	13	7	20
III	23	11	34
IV	2	0	2
HR			
Positive	12	6	18
Negative	28	12	40
Her 2			
Positive	11	5	16
Negative	29	13	42
TNBC	6	3	9
Chemotherapy			
Neoadjuvant	8	4	12
Adjuvant	32	14	46

Simulation

Positioning and CT Scanning

All patients underwent simulation using the GE Optima 580W® dedicated CT simulator. Patients were positioned supine using a 100-degree inclination breast board (Macromedics Medical Solution®) with both arms raised above the head in a comfortable and reproducible position with heads turned to the opposite side of the affected breast as much as they could. Marking over the chest wall was done for daily set up reproducibility. Axial images of area of interest in patients were acquired.

Clinical Target Volume (CTV) and OAR Delineation

Target volume delineation and OAR contouring were done as per the Radiotherapy Oncology Group (RTOG) guidelines. The planning target volume (PTV) to CTV margin was taken 5 mm isotropically for all cases.

Dose Prescription

All cases were planned for 40 Gy in 15 fractions to PTV, with a daily fraction size of 2.67 Gy, administered five days a week over a period of three weeks.

Treatment Planning Techniques

For each patient, VMAT and IMRT plans were generated using the same Monaco TPS and optimized using the Monte Carlo algorithm. One hundred sixteen plans were generated and evaluated for dose-volume histogram (DVH) parameters.

For VMAT plans two partial arcs in clockwise (CW) and counterclockwise (CCW) directions were used, while IMRT plans were generated using five to nine fields (mostly seven) with a tangential field setup. Inverse planning was performed to achieve optimal PTV coverage (i.e., 95% of PTV volume should receive minimum 95% of prescribed dose), and the hotspot should be less than 2 cc and outside the PTV. Doses received by OARs were kept well within their tolerance values.

The treatment plans were administered using the Elekta Versa HD®, which featured the Agility® multi-leaf collimator (MLC). To increase the skin surface dose, a 5-mm thick bolus was applied over the chest wall during half of the treatment. Weekly kilovoltage cone-beam computerized tomography (KV-CBCT) imaging was conducted for setup and verification purposes, with tolerance limits set at 5 mm.

Plan assessment

For the PTV, a number of dosimetric parameters, including V95% and V107% as well as D2, D50, and D98 were examined. Additionally, the homogeneity index (HI), conformity index (CI), and Dmean were examined.

For the ipsilateral lung Dmean and Dmax, as well as V5, V10, V20, V30, and V40 (representing the volume of the ipsilateral lung receiving 5 Gy, 10 Gy, 30 Gy, and 40 Gy, respectively) were evaluated.

V5 and V10 were assessed for the contralateral lung. For the contralateral breast V2 and V5 as well as Dmean and Dmax were evaluated. Finally, for the heart, Dmean, V5, V10, V20, and V35 (representing the volume of the heart receiving 5 Gy, 10 Gy, 20 Gy, and 35 Gy, respectively) were analyzed.

Statistical analysis

Using SPSS version 22 (IBM Corp., Armonk, NY), a statistical analysis was performed. The parameters of the VMAT and IMRT plans were contrasted using the paired t-test. The threshold for statistical significance was set at 5%. The chi-square test was used to compare the dosimetric profiles of the OARs, which were provided as frequencies and percentages.

## Results

Target volume coverage

Every plan in our analysis complied with the requirement for V95 > 95% target volume coverage. With the exception of the Dmean and conformance index, we found no discernible variations in most target volume metrics between VMAT and IMRT plans for right-sided breast cancer (CI). While the Dmean was much lower in IMRT plans, the CI was significantly better in VMAT plans (p = 0.004). Additionally, for patients with right-sided breast cancer, VMAT plans required fewer total MUs. Both VMAT and IMRT plans had equivalent target volume parameters in cases of left-sided breast cancer; however, VMAT plans were discovered to be more conformal (p = 0.03) (Table 2).

**Table 2:** Comparison of PTV DVH parameters in VMAT and IMRT plans PVT - planning target volume, DVH - dose-volume histogram, VMAT - volumetric-modulated arc therapy, IMRT - Intensity-modulated radiation therapy

Site of the disease	Right Sided			Left Sided		
Parameters	VMAT (mean ±SD)	IMRT (mean ±SD)	p value	VMAT (mean ±SD)	IMRT (mean ±SD)	p value
V95 %	98.38±1.3	96.52±4.0	0.07	97.32±1.22	97.35±1.07	0.48
V107%	0.22±0.49	0.42±0.74	0.21	1.45±3.93	1.69±3.06	0.35
D2(Gy)	41.95±0.36	42.02±0.45	0.32	42.10±0.64	42.51±1.49	0.18
D98(Gy)	38.3±0.75	37.77±1.05	0.07	37.67±0.71	37.61±0.70	0.42
D50(Gy)	40.73±0.27	40.58±048	0.12	40.69±0.45	40.74±0.59	0.4
Dmean	40.64±0.20	40.43±0.51	0.04	40.57±0.43	40.60±0.59	0.43
Dmax	43.83±0.47	44.06±0.90	0.21	44.09±0.88	44.73±2.47	0.19
HI	1.07±0.02	1.08±0.03	0.09	1.08±0.02	1.09±0.03	0.19
CI	0.71±0.12	0.65±0.09	0.04	0.72±0.07	0.66±0.06	0.03
Avg. total MU	923.03±65	973.74±173	0.16	1022.79±113	1024.38±133	0.49

Dose to lungs

For right-sided breast cancer, VMAT plans showed considerably lower V20, V30, and V40 than IMRT plans (p < 0.05); however, VMAT plans exhibited greater V5 and V10 of the ipsilateral lung compared to IMRT plans. For the ipsilateral lung, there were no appreciable variations in Dmax and Dmean between the two methods. Both V5 and V10 were considerably lower in IMRT plans for the contralateral lung (p < 0.05). In comparison to IMRT plans, the VMAT plan for left-sided breast cancer exhibited significantly larger V5 and V10 of the ipsilateral lung, but significantly lower V20, V30, and V40 (p < 0.05). For the ipsilateral lung, there were no appreciable variations in Dmax and Dmean between the two methods. For VMAT plans, the Dmax was significantly lower. Both V5 and V10 were noticeably lower in IMRT plans for the contralateral lung (p < 0.05).

Dose to contralateral breast

In all cases, both VMAT and IMRT plans achieved a mean dose of 5 Gy to the contralateral breast. Dmax was lower in VMAT plans whereas the Dmean, V2, and V5 were much lower in IMRT plans.

Dose to heart

Both planning strategies were successful in achieving a V20 Gy to the heart of less than 10% for right-sided breast cancer, which was not possible in cases of left-sided breast cancer. In IMRT designs, the heart's mean dosage was considerably lower. Between 5 Gy and 35 Gy, IMRT plans also seemed to spare the heart more effectively. While V20 and V35 were more successfully attained by VMAT plans, the V5 was much lower for IMRT plans.

Dose to spinal cord

The maximum dose achieved at the spinal cord was significantly lower in IMRT plans, regardless of the laterality of the disease (p < 0.05) (Table 3).

**Table 3:** Comparison of OAR DVH parameters in VMAT and IMRT plans OAR - organ at risk, DVH - dose-volume histogram, VMAT - volumetric-modulated arc therapy, IMRT - Intensity-modulated radiation therapy

Site of the disease	Right Sided			Left Sided		
Structure	VMAT (mean ±SD)	IMRT (mean ±SD)	p value	VMAT (mean ±SD)	IMRT (mean ±SD)	p value
Ipsilateral lung						
V5	79.9±13.97	66.34±15.60	<0.05	82.11±12.24	75.73±14.3	0.05
V10	50.02±9.25	49±11.86	0.36	52.15±8.41	48.58±7.20	0.05
V20	30.25±3.87	36±10.68	0.01	29.98±4.59	34.6±6.28	<0.05
V30	19.12±3.36	26.20±8.83	<0.05	17.78±2.87	24.14±6.37	<0.05
V40	2.74±1.71	4.89±3.60	0.02	2.42±1.43	5.90±3.17	<0.05
D mean	15.33±1.70	16.09±3.53	0.16	15.45±1.69	16.25±2.05	0.07
D max	42.92±0.71	42.97±0.86	0.43	42.92±0.93	43.71±1.32	0.03
Contralateral lung						
V5 (C/L)	9.62±5.51	2.77±4.27	<0.05	22.92±16.36	8.06±9.52	0.02
V10 (C/L)	0.31±0.47	0.04±0.08	0.03	2.53±3.74	0.37±0.72	0.04
Contralateral breast						
D max	16.29±7.58	25.93±10.14	<0.05	17.32±7.75	29.55±11.05	<0.05
D mean	2.59±0.52	1.74±0.62	<0.05	3.56±1.04	2.46±1	<0.05
V2	56.32±20.43	17.39±14.80	<0.05	75.05±21.44	27.27±20.29	<0.05
V5	6.32±3.86	4.33±4	<0.05	18.07±14.27	9.12±7.36	0.01
Heart						
D mean	5.87±2.22	3.41±1.72	<0.05	10.66±1.97	10.66±3.21	0.5
V5	44.36±24.3	20.60±21.9	0.01	78.68±9.97	56.41±18.24	<0.05
V10	13.96±10.82	3.72±5.44	<0.05	35.91±12.23	33.46±14.52	0.28
V20	1.76±2.07	0.52±0.99	0.01	12.79±5.25	18.73±8.73	0.01
V35	0.02±0.04	0.01±0.03	0.12	1.89±1.41	5.22±2.99	<0.05
Spinal Cord						
D max	19.87±6.51	11.06±6.35	<0.05	21.35±3.62	12.61±5.30	<0.05

## Discussion

In comparison to VMAT or IMRT, traditional 3D-CRT frequently produces worse conformance and homogeneity, according to a review of the literature by Balaji et al. [16] While IMRT and VMAT boost the target's dosage conformity, they do so at the expense of extending the low dose bath to the breast and lung on the contralateral side, which raises the risk of secondary malignancy.

The dosimetric properties of various radiation modalities, such as VMAT, IMRT, and 3D-CRT, have been compared in a number of studies. The majority of these studies, however, are restricted to left-sided breast malignancies following breast conservation surgery and used conventional radiation fractionation. Right- and left-sided breast cancer patients who had mastectomy and were treated with either VMAT or IMRT were included in our study.

Zhang et al. [17] reported that VMAT performed better than IMRT in terms of dosimetric parameters for both the PTV and OARs. In our research, we discovered that PTV coverage across VMAT and IMRT schemes was comparable. However, we found that, when compared to IMRT, VMAT demonstrated better dose homogeneity and noticeably better dose conformity in both right- and left-sided breast cancer plans. Moreover, we discovered that in IMRT designs for right-sided breast tumors, the Dmean for PTV was much lower. In contrast, we found no appreciable variations in the other assessed characteristics.

Compared to VMAT, IMRT treatment plans often require more MUs and longer delivery times. Increased radiation leakage and scatter due to longer treatment times and more MUs may hinder tumor cell repopulation and repair [9,18]. Shorter treatment periods brought on by the use of fewer MUs in VMAT plans may minimize the incidence of long-term secondary malignancies in patients [17,19] and may also lower the risk of intrafractional motion and scattered dosage. In contrast, Sakthivel et al.'s study found no connection between the number of MUs and excess absolute risk (EAR) [20].

In our study, VMAT plans delivered slightly lower MU to the right-sided breast cancer patients (923 ± 65 vs 973 ± 173), whereas the MUs were comparable in both techniques for left-sided breast cancers. This finding was in accordance with most of the studies [11,19,21], but contrary to the study by Sakthivel et al. [20], where MUs in IMRT were much lesser than VMAT.

A serious complication that could happen to breast cancer patients following radiotherapy is radiation pneumonitis. The DVH lung parameters V5Gy, V10Gy, and V20Gy are significant for assessing the risk of radiation pneumonitis [22-24]. For every 10% increase in V10Gy, radiation-induced pneumonitis incidence rises by 10%, according to Willner et al. [25]. Regardless of the laterality of the tumor, our investigation demonstrated that IMRT plans had noticeably lower dose parameters for the ipsilateral lung (including V5Gy and V10Gy) than VMAT plans. Nevertheless, with V20Gy (p < 0.05), V30Gy (p < 0.05), and V40Gy (p < 0.05), VMAT plans produced better outcomes. In terms of low-dose distribution (V5Gy and V10Gy) for contralateral lung dose, IMRT was found to be superior. Our results were in line with the research by Ma et al. [7], which found that lung V5Gy and V10Gy for five-field IMRT plans were superior to those for two-partial-arc VMAT plans. In agreement with our work, Lai et al. [5] likewise showed reduced V20Gy and higher V5Gy via VMAT.

Popescu et al. [26] found that limiting the Dmean of the contralateral breast below 3.2 Gy can greatly lower the incidence of subsequent malignancies after radiation. Regardless of the laterality of the primary disease, the Dmean of the contralateral breast in our study was significantly lower in IMRT plans (1.74±0.62 for right-sided tumors and 2.46±1 for left-sided tumors) compared to VMAT plans (2.59±0.52 for right-sided tumors and 3.56±1.04 for left-sided tumors). Moreover, the lowest Dmax of the contralateral breast was found in VMAT plans (p = 0.05).

In the modern period, radiation was used to treat both left- and right-sided breast cancer, and the PASSOS heart research reported no difference in cardiac morbidity [27]. According to Darby et al.’s hypothesis, the incidence of coronary events rose by 7.4% for every Gy of mean heart dosage [28]. For right-sided tumors in our investigation, the Dmean of the heart was much lower in IMRT designs than it was for left-sided tumors. The work by Zhao et al. [29] demonstrated that IMRT was superior at attaining a lower Dmean whereas VMAT was superior at achieving V20Gy and V40Gy doses for the heart. The findings of our investigation and the study by Zhao et al. [29] were in agreement. For right-sided breast malignancies, IMRT plans demonstrated many superior results for V5Gy, V10Gy, V20Gy, and V35Gy, while VMAT plans demonstrated significantly lower V20Gy and V35Gy for left-sided breast cancers. The variability of patients and types of operation are responsible for the inconsistent results in earlier studies involving various DVH parameters.

This research has several restrictions. First off, since it was a dosimetric trial, we are unable to directly compare it to actual clinical outcomes. Second, there were no techniques in place to regulate breathing patterns, which would have had an impact on the actual dosage delivered to the target volume and OARs during breast radiation.

## Conclusions

Our study demonstrates that although the VMAT and IMRT plans had similar target area coverage, VMAT exhibited better conformity. However, compared to IMRT plans, VMAT plans also led to higher dose spillage in adjacent normal tissues. In contrast, IMRT strategies successfully reduced radiation exposure to a number of organs that were vulnerable to both left- and right-sided breast cancers. Particularly, at larger doses, VMAT was more effective at minimizing radiotherapy doses to the heart and ipsilateral lung.

Hence, the best radiation strategy for breast cancer should be decided according to the case, taking tumor lateralization and the risk-benefit trade-off into consideration. To get the best results from your treatment, it is critical to thoroughly weigh the advantages and disadvantages of each technique.
